# Carcinome parathyroïdien géant: difficultés diagnostiques et stratégies thérapeutiques

**DOI:** 10.11604/pamj.2017.26.211.8770

**Published:** 2017-04-19

**Authors:** Ilias Benchafai, Leila Afani, Noureddine Errami, Bouchaib Hemmaoui, Hassan Errihani, Fouad Benariba

**Affiliations:** 1 Service d’ORL, Hôpital Militaire d’Instruction Mohammed V, Rabat, Maroc; 2 Service d’Oncologie, Institut National d’Oncologie My Abdellah, Rabat, Maroc

**Keywords:** Hyperparathyroïdie, hypercalcémie, carcinome parathyroïdien, traitement, récidive, Hyperparathyroidism, hypercalcemia, parathyroid carcinoma, treatment, recurrence

## Abstract

Le carcinome parathyroïdien est une tumeur maligne très rare responsable de 0,4 à 5,2% des hyperparathyroïdies. Son diagnostic clinique est difficile et son traitement doit être codifié. La chirurgie reste le seul traitement curatif. Nous rapportons le cas d’une patiente suivie pour hypercalcémie maligne qui a révélé un carcinome parathyroïdien, elle a été opérée et a présenté 3 mois plus tard une récidive ganglionnaire. Vu l’absence d’autres localisations secondaires un curage ganglionnaire bilatéral suivi d’une chimiothérapie ont été instaurés. Le carcinome parathyroïdien est souvent suspecté devant des critères biologiques radiologiques et surtout macroscopiques peropératoires mais le diagnostic de certitude reste histopathologique. Le seul traitement curatif repose sur la chirurgie et la place d’un traitement adjuvant reste encore à établir.

## Abstract (en)

Parathyroid carcinoma is a very rare malignancy responsible for 0.4 to 5.2% of hyperparathyroidism. Clinical diagnosis is difficult and treatment should be codified. Surgery is the only curative treatment. We report the case of a female patient treated for malignancy-associated hypercalcemia revealing parathyroid carcinoma. The patient underwent surgery; after three months she developed lymphatic recurrence. Given the absence of other secondary involvement, bilateral lymph node dissection followed by chemotherapy was performed. Parathyroid carcinoma is often suspected on the basis of biological, radiological and especially intraoperative macroscopic criteria, but definitive diagnosis is made histopathologically. Surgery is currently the only curative treatment and the role of adjuvant therapy is to establish.

## Introduction

Le carcinome parathyroïdien est une tumeur très rare avec une prévalence estimée à 0,005% de tous les cancers. Il est responsable de 0,4 à 5,2% des causes des hyperparathyroïdies [1]. C’est une maladie d’évolution lente dont le pronostic est plus lié aux complications de l’hypercalcémie. La prise en charge du carcinome parathyroïdien pose un problème diagnostic et thérapeutique. La distinction entre adénome et carcinome parathyroïdien reste difficile. Le seul traitement curatif repose sur la chirurgie et la place d’un traitement adjuvant reste encore à établir [2]. A travers cette observation, nous développons les principales caractéristiques cliniques histologiques et thérapeutiques de cette entité clinique.

## Patient et observation

**Figure 1:**
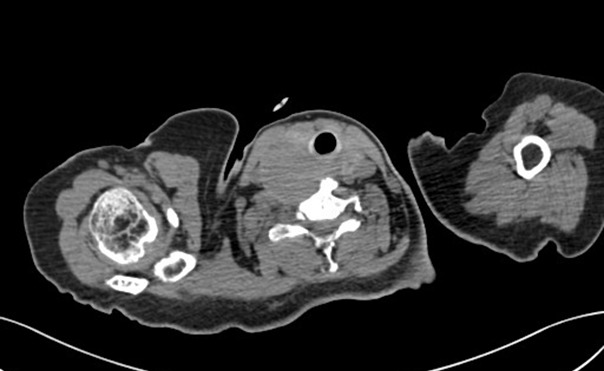
Coupe tomodensitométrique axiale montrant une masse parathyroïdienne droite au contact de la thyroïde, de la trachée et du plan prévertebrale

**Figure 2:**
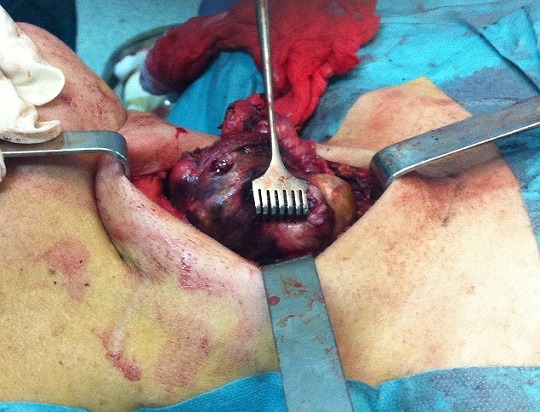
Aspect peropératoire du carcinome parathyroïdien et du lobe thyroïdien homolatéral

**Figure 3:**
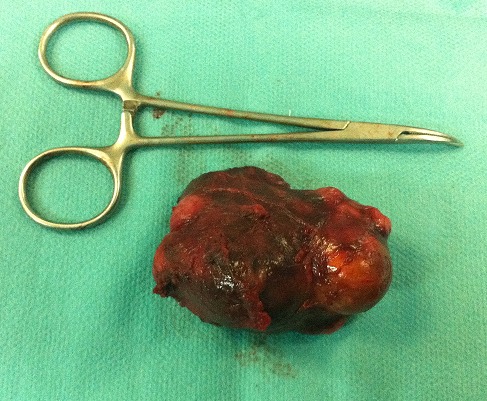
Pièce opératoire

**Figure 4:**
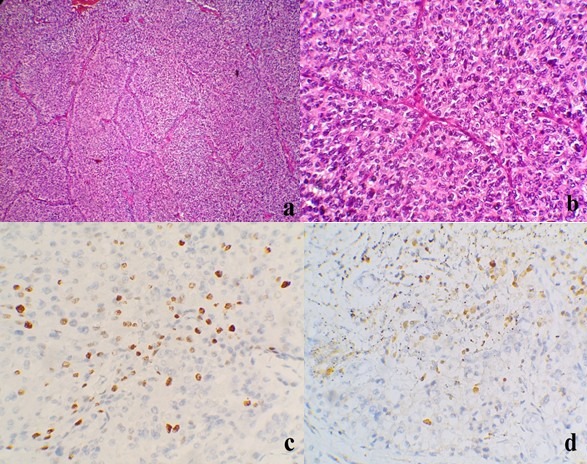
(A) prolifération tumorale faite de nappes et de massifs, avec un stroma grêle vasculaire. (HE, Gx50); (B) les cellules tumorales sont pourvues d’un cytoplasme éosinophile granulaire et de noyaux arrondis ou ovoïdes, légèrement hyperchromatiques. (HE, Gx400) (C) l’index de prolifération ki-67 est estimé à environ 15%. (Gx400); (D) immunomarquage positif faible et focal des cellules tumorales à l’anticorps anti-bcl2. (Gx400)

**Figure 5:**
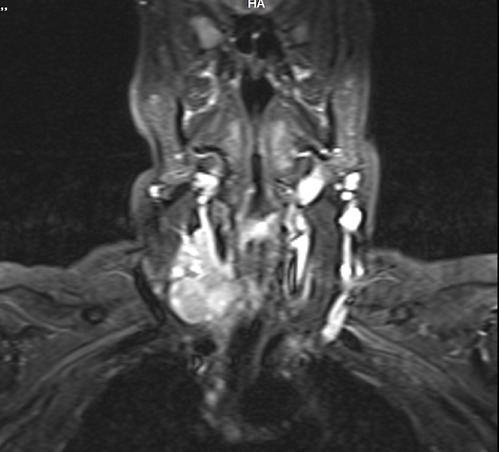
IRM en coupe coronale montrant la récidive ganglionnaire jugulo-carotidienne droite

Il s’agit d’une patiente de 52 ans; sans antécédents pathologiques notables; qui a été admise dans notre formation dans un tableau d’hypercalcémie aigue. L’histoire de sa maladie remontait à deux mois auparavant où elle a présenté une asthénie, des douleurs osseuses diffuses, un syndrome polyurique polydypsique et une constipation le tout évoluant dans un contexte d’altération de l’état général. L’examen clinique retrouvait une masse latéro-cervicale droite indolore de consistance dure mesurant 6cm de diamètre. Le bilan biologique préopératoire avait objectivé une hypercalcémie à 172mg/l, une insuffisance rénale, une hyperparathyroïdie avec un taux de parathormone à 555pg/ml et un syndrome inflammatoire modéré (Tableau 1). Le bilan de retentissement de l’hypercalcémie a révélé: sur le plan cardiaque: un raccourcissement de l’espace QT à l’électrocardiogramme, sur le plan rénal: une insuffisance rénale avec microlithiase rénale à l’échographie et sur le plan digestif : une gastrite congestive et une pancréatite stade A. Devant cette hypercalcémie maligne menaçante, un traitement a été instauré, basé sur une hydratation massive par du sérum salé avec administration de diurétiques et de biphosphonates. Une échographie cervicale a objectivé une volumineuse masse parathyroïdienne droite, hypoéchogène hétérogène avec des zones de nécrose et des calcifications, mesurant 67x38x49mm de diamètre et une glande thyroïde siège de 2 nodules polaires inférieurs gauches avec absence d’adénopathies cervicales. La scintigraphie au Tc-99m sestamibi a montré: une fixation parathyroïdienne droite inferieure. La tomodensitométrie cervicale a montré: la présence d’une masse parathyroïdienne droite inferieure mesurant environ 6cm et qui est en contact intime avec la thyroïde, la trachée et le plan para vertébral (Figure 1). Après contrôle du taux de calcémie qui est revenu à 100mg/l. Un traitement chirurgical a été indiqué. Le geste chirurgical a consisté en l’exérèse d’une masse ovoïde jaunâtre retro thyroïdienne droite mesurant environ 6cm de grand axe adhérente au lobe thyroïdien homolatéral et à la trachée. Malignité suspectée le geste chirurgical a été complété par une thyroïdectomie totale et un curage ganglionnaire de la chaine récurrentielle homolatérale (Figure 2, Figure 3). L’étude histologique a décrit: au niveau parathyroïdien: la pièce mesure 6x5x4cm, les coupes analysées ont montré une prolifération tumorale faite de cordons et de massifs, les cellules ont un noyau arrondi à ovoïde légèrement hyperchromatique, le cytoplasme et éosinophile et granulaire, le stroma est grêle et vasculaire. Les recoupes ont montré la présence d’une effraction capsulaire focale et de quelques emboles vasculaires. Le complément immuno-histochimique a montré: l’anti-composanteBcl2: marquage faible et focale des cellules tumorales. L’anti-composante Ki67: faible estimée à 15% (Figure 4). Au niveau thyroïdien: goitre multi-hétero-nodulaire sans signes histologiques de malignité. Au niveau ganglionnaire: absence d’atteinte ganglionnaire N0. Le bilan d’extension: TDM thoraco-abdomino-pelvienne a éliminé l’existence d’une lésion secondaire à distance. Les suites opératoires étaient simples et marquées par une baisse des taux de calcémie et de parathormonémie (Tableau 1). Le suivi à 3 mois a révélé l’apparition de poly adénopathies cervicales jugulo-carotidiennes bilatérales et médiatisnales (Figure 5). La TDM thoraco-abdomino-pelvienne a éliminé l’existence d’une lésion secondaire à distance. La patiente a été reprise chirurgicalement et a bénéficié d’un curage ganglionnaire jugulo-carotidien fonctionnel bilatéral et un curage ganglionnaire médiastino-reccurentiel bilatéral. L’étude histologique était en faveur de métastases ganglionnaires d’un carcinome parathyroïdien. Ensuite la patiente a reçu 6 cures de chimiothérapie à 3 semaines d’intervalle à base de dacarbazine 875mg /m^2^. Le suivi à 6 mois n’a pas objectivé de récidives.

**Tableau 1:** Valeurs des données biologiques avant et après chirurgie

Données biologiques	Préopératoires	Postopératoires
Créatinine	32 mg/l	36 mg/l
Calcémie	172 mg/l	100 mg/l
phosphore	43 mg/l	34 mg/l
PTH	555 pg/l	48 pg/l

## Discussion

Notre cas clinique traite la problématique diagnostique et thérapeutique que pose la découverte d’une hyperparathyroïdie. Les principales étiologies sont les adénomes parathyroïdiens; les hyperplasies parathyroïdiennes et le cancer parathyroïdien. Le tableau clinique symptomatique nous a fait suspecter une pathologie maligne. Les tumeurs bénignes des parathyroïdes se présentent souvent sous forme pauci ou asymptomatique contrairement à la plupart des cancers parathyroïdiens qui sont sécrétant et se présentent souvent dans un tableau d’hypercalcémie profonde [3]. Le carcinome parathyroïdien est une tumeur rare avec une prévalence estimée à 0.005% de tous les cancers .le premier cas a été décrit en 1909 par Quervain [1-4]. L’âge de survenue se situe entre 45 et 59 ans. Il n’y a pas de prépondérance féminine comme dans les adénomes parathyroïdiens [4]. L’étiopathogénie reste inconnue. Des cas ont été rapportés dans le cadre de syndromes héréditaires [5]: les néoplasies endocriniennes multiples de type I ou de type IIA [6], le syndrome d’hyperparathyroïdie primaire avec tumeur maxillaire et l’hyperparathyroïdie primaire néonatale. Le carcinome parathyroïdien non métastatique constitue un véritable problème diagnostic. Il est particulièrement difficile de différencier entre tumeurs parathyroïdiennes bénignes (adénomes) et carcinomes parathyroïdiens. Ces derniers se font suspecter devant des symptômes d’hypercalcémie sévère due à l’hyperparathyroïdie primitive [1, 4, 7]. En fait cette hypercalcémie menaçante est responsable d’une symptomatologie polymorphe et plus prononcée que celle des adénomes parathyroïdiens. Elle est faite dans la plupart des cas de signes ostéo-articulaires : douleur osseuse, ostéoporose, chondrocalcinose articulaire et fractures pathologiques, associés souvent à des signes rénaux : syndrome polyurodipsique, colique nephretique, lithiase rénale voire insuffisance rénale au stade d’hémodialyse. Les signes digestifs ne sont pas rares : anorexie, nausées, douleurs abdominales, constipation, ulcères gastroduodénaux et pancréatites. Les manifestations cardiovasculaires sont fréquentes: troubles du rythme, et hypertension artérielle. Aussi une altération de l’état général, une asthénie physique ou psychique ou bien un syndrome dépressif peuvent révéler la maladie [4]. Ces signes étant non spécifiques retardent souvent le diagnostic. 30% à 70% des patients atteints de carcinome parathyroïdien présentent une masse latérocervicale palpable, ferme et extra-thyroïdienne, mesurant entre 3 et 6 cm de diamètre[2], ce qui contraste avec les tumeurs bégnines des parathyroïdes qui sont généralement non palpables. La présence d’adénopathies cervicales ou de paralysie laryngée sont en faveur de malignité. Environ 33% des patients ont des métastases à distance pulmonaires, hépatiques et osseuses [7].

Les taux sériques de calcium et de parathormone sont significativement plus élevés que lors des tumeurs bégnines des parathyroïdes, avec un calcium sérique supérieur à 14mg/dl et une parathormone égale à 3 à 10 fois la limite supérieure de la normale. D’autres marqueurs biologiques tels que les phosphatases alcalines sont aussi élevés. Devant ces signes clinico-biologiques d’hyperparathyroïdies primaires, l’imagerie préopératoire doit systématiquement comporter une échographie cervicale qui objectivera une masse cervicale retrothyroïdienne paratrachéale hypoéchogène, de contours irréguliers avec des signes d’invasion des structures adjacentes [2]. Cette échographie sera complétée par une scintigraphie au Tc99m-sestamibi, sensible à 91%, qui permet de mettre en évidence la présence d’une tumeur parathyroïdienne (hyperfixation) et de la localiser [2]. Ces deux examens de référence confirment la nature parathyroïdienne de la masse cervicale et recherche la présence d’éventuelles adénopathies cervicales. En cas de forte présomption de malignité, une IRM cervicale peut étudier l’extension locorégionale et mettre en évidence une masse cervicale en hypersignal en T1 et en T2, pluricentimétrique au niveau de la face postérieure de la thyroïde. Une TDM thoraco-abdomino-pelvienne recherchera des métastases à distance [8]. Il n’existe pas de classification TNM vu la rareté de cette pathologie. Donc seule l’étude histologique permet un diagnostic de certitude de carcinome parathyroïdien. La cytoponction à l’aiguille fine est à proscrire car outre la difficulté d’affirmer la nature maligne des échantillons cytologiques, elle peut provoquer une dissémination tumorale.Cependant elle peut être utile pour différencier entre une récidive tumorale et de la fibrose [9]. Le diagnostic peropératoire de carcinome parathyroïdien n’est pas facile. Macroscopiquement c’est une masse solide, de consistance dure, polylobée, encapsulée, de couleur grise à blanchâtre, mesurant plus de 3cm de diamètre, pesant plus de 12g et qui adhère étroitement au lobe thyroïdien ou aux tissus adjacents (muscles sous-hyoïdiens, nerf récurrent, trachée, œsophage,…). Microscopiquement , Schantz et Castelman ont défini des critères pour le diagnostic du carcinome parathyroïdien, ce sont: la présence de figures de mitose, l’architecture trabéculaire ou en rosette, la présence de bandes fibreuses irradiant à partir de la capsule, l’invasion capsulaire et les emboles vasculaires [10]. Récemment des techniques d’immunohistochimie utilisant des anticorps monoclonaux, PCNA et Ki67, ont été étudiées et peuvent être utilisées comme facteur pronostic d’agressivité.

Avant toutes thérapeutiques, une préparation médicale s’avère nécessaire, elle permet de juguler l’urgence métabolique (crise hypercalcémique) et consiste en une réhydratation massive intraveineuse par du sérum salé isotonique et une administration de diurétiques de l’anse de Henlé. Les biphosphonates , qui bloquent la résorption osseuse, peuvent être associées. Parfois des séances d’hémodialyse pour restaurer la calcémie peuvent s’avérer nécessaires [7]. Le traitement de choix reste chirurgical. Après constatation peropératoire des critères de malignité; déjà cités; le chirurgien doit être amené à exécuter une résection en bloc de la glande tumorale et du lobe thyroïdien homolatéral (parathyroïdectomie + loboisthmectomie homolatérale), en respectant le nerf récurrent s’il n’est pas envahis, ainsi qu’un curage ganglionnaire médiastino-récurrentiel homolatéral de principe (groupe VI). Si présence d’adénopathies métastatiques, un curage ganglionnaire cervical homolatéral fonctionnel sera réalisé. Une attention particulière doit être portée à la capsule tumorale évitant sa rupture et donc la dissémination tumorale, permettant ainsi une meilleure chance de guérison [7, 9, 10]. Le carcinome parathyroïdien est radiorésistant. Il n’existe pas de protocole de radiothérapie prédéfini. Cependant pour certains auteurs la radiothérapie externe post-opératoire à la dose de 40 à 70 Gy peut être proposée en cas d’envahissement locorégional et elle semble diminuer le risque de récidive locale [7, 9]. La chimiothérapie est généralement inefficace dans le traitement du carcinome parathyroïdien.De nombreuses drogues ont été utilisées comme la dacarbazine, la vincristine, l’actinomycineD et l’adriamycine, en monothérapie ou combinées avec le 5 Fluoro-uracile et le cyclophosphamide [7]. La chimiothérapie , dont les protocoles ne sont pas encore établis , ne peut se concevoir que pour les tumeurs polymétastatiques. La radiothérapie et la chimiothérapie peuvent être proposées comme traitement palliatif des patients présentant un carcinome parathyroïdien inopérable ou métastatique [10]. Le suivi thérapeutique est facilement réalisé par le taux sérique de la calcémie et de la parathormonémie. Le taux de récidive locale même tardive est estimé à 30 à 70%. Le taux de métastases ganglionnaires ou à distance surtout pulmonaires ou osseuses est estimé à 30% des cas [8]. La survie globale à 5 ans est de 85% et à 10 ans est de 49 à 77% [2, 7].

## Conclusion

Le carcinome parathyroïdien est une tumeur maligne rare hypersecrétante et dont le diagnostic reste difficile. Il est suspecté devant un syndrome d’hyperparathyroïdie primaire sévère puis par l’aspect de la glande tumorale en peropératoire et son extension aux tissus adjacents. Le diagnostic est confirmé par l’étude histologique grâce à des techniques récentes d’immunohistochimie. Le traitement de choix est chirurgical et il est associé à une radiothérapie externe dans certains cas avancés. La chimiothérapie n’a pas encore fait preuve d’efficacité. La surveillance post-thérapeutique se base sur le dosage de la calcémie et de la parathormonémie.
